# Retraction Note: Decreased neuroinflammation and increased brain energy homeostasis following environmental enrichment after mild traumatic brain injury is associated with improvement in cognitive function

**DOI:** 10.1186/s40478-022-01380-1

**Published:** 2022-05-17

**Authors:** Teresita L. Briones, Julie Woods, Magdalena Rogozinska

**Affiliations:** 1 Department of Adult Health, Cohn Bldg, Rm 344, Wayne State University, 5557 Cass Avenue, Detroit, MI 48202 USA; 2 Department of Biobehavioral Health Science, University of Illinois at Chicago, Chicago, IL 60612 USA

## Retraction Note: Acta Neuropathologica Communications (2013) 1:57 10.1186/2051-5960-1-57

The Editor-in-Chief has retracted this article because of significant concerns regarding a number of Figures presented in this work. After publication, duplicated western blot bands were identified in Figs. 2b (AMPK lanes 2&3) and 3b (uM1CK lanes 1&2), as well as Fig. 3a (beta-actin lanes 1 and 2). The Editor-in-Chief therefore no longer has confidence in the integrity of the data in this article.

Magdalena Rogozinska has stated that she was not involved in the preparation of the manuscript.

Teresita L. Briones agrees to this retraction. Magdalena Rogozinska has not responded to any recent correspondence from the Publisher to indicate they agree or disagree with this Retraction Notice. The Publisher has not been able to contact Julie Woods.

